# Understanding cell signalling systems: paving the way for new therapies

**DOI:** 10.1098/rsta.2013.0155

**Published:** 2015-03-06

**Authors:** E. Yvonne Jones

**Affiliations:** Division of Structural Biology, Wellcome Trust Centre for Human Genetics, University of Oxford, Roosevelt Drive, Oxford OX3 7BN, UK

**Keywords:** cell guidance cue, ligand–receptor complex, semaphorin, plexin, neuropilin

## Abstract

The cell-to-cell signalling mechanisms of multi-cellular organisms orchestrate human development during embryogenesis and control homeostasis in adult tissues. These are mechanisms vital to human health and perturbation of cell-to-cell signalling is a contributing factor in many pathologies including cancer. The semaphorin cell guidance cues and their cognate plexin receptors exemplify a cell-to-cell signalling system for which insights into mechanistic principles are emerging. X-ray crystallographic data from Diamond beam lines have enabled us to probe the inner workings of semaphorin–plexin signalling to atomic-level resolutions. Importantly, we can complement protein crystallographic results with biophysical and cellular studies to dovetail structural information with functional impact. The signature seven-bladed *β* propeller ‘sema’ domain of the semaphorins forms a dimer; in contrast the equivalent domain in the plexins is monomeric. The generic architecture of a semaphorin–plexin complex is characterized by the dimeric semaphorin cross-linking two copies of the plexin receptor. For specific family members, the co-receptor neuropilin serves to bolster this architecture, but in all cases, the dimeric interaction lies at the core of the ligand receptor complex, providing the essential trigger for signalling.

## 1. Introduction

**Figure 1.**
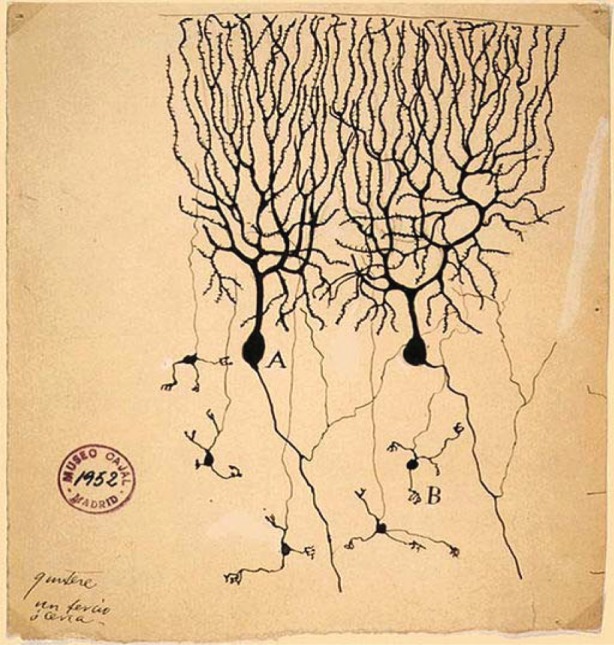
Drawing of Purkinje cells (A) and granule cells (B) from pigeon cerebellum. Credit: Santiago Ramón y Cajal, 1899. Instituto Santiago Ramón y Cajal, Madrid, Spain. (Online version in colour.)

The Instituto Santiago Ramón y Cajal in Madrid has in its keeping a seminal series of ink drawings made towards the end of the nineteenth century. In these drawings, Cajal records observations he made using the cutting-edge microscopy techniques of his day. The drawings show the intricate networks formed by the dendrites and axons extending from cells of the nervous system in, for example, a pigeon cerebellum (figure 1). The complexity of this cellular organization led Cajal to the hypothesis that this organization must be the result of guidance mechanisms provided by chemical gradients. A century later, in the 1990s, four families of protein molecules were characterized as the providers of guidance cues during neuronal development: the netrins, slits, ephrins and semaphorins [1]. The wiring of nervous systems, ranging from fly to human, requires cell surface receptor-based signalling systems to guide the growing neurites to their correct locations. Indeed, it is now apparent that the development and homeostasis of tissues throughout the human body is founded on cell guidance systems. The common theme running through the mechanisms of action of these systems is that binding of the extracellular ligand, a secreted or cell attached guidance cue, to a cell surface receptor triggers intracellular signals which cause localized changes in the cytoskeleton. This review focuses on recent insights into the atomic-level workings of the semaphorin system of guidance cues; results, over 100 years after Santiago Ramón y Cajal, of the cutting-edge technology of today at the Diamond Light Source.

## 2. The semaphorins

The semaphorins were first identified in the grasshopper [2], and, with the discovery in chickens and humans of proteins sharing the same, signature, approximately 500 residue N-terminal ‘sema’ domain sequence, this evolutionarily conserved family was defined [3,4]. The semaphorins constitute one of the major families of cell guidance cues and with their cognate receptors, the plexins [5], mediate repulsive and, less commonly, attractive guidance responses in diverse physiological processes ranging from cell migration, angiogenesis and neural connectivity to regulation of immune responses [6–8]. Conversely, dysfunctional semaphorin–plexin signalling is implicated in tumour progression [9]. The extracellular interactions mediated by the sema domain are central to the biology of the semaphorin signalling system.

## 3. The dimeric nature of the semaphorin sema domain

**Figure 2.**
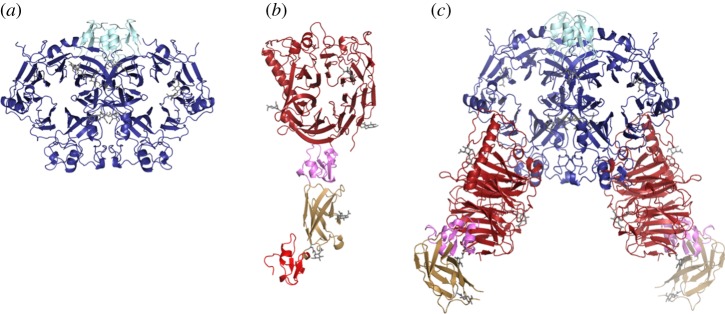
Ribbon representations of semaphorin, plexin and semaphorin–plexin complex structures. (*a*) mSema6A_*ecto*_ (sema domain in blue and PSI domain in cyan). (*b*) mPlxnA2_1−4_ (sema domain in red, first PSI domain in pink, Ig-like IPT domain in wheat and second PSI domain in crimson). (*c*) The mPlxnA2_1−4_–mSema6A_ecto_ complex (colours as in previous panels). The second (C terminal) PSI domain is included in the unliganded mPlxnA2_1−4_ crystal structure but lacks well-ordered electron density in the complex crystal structure.

In 2003, my laboratory (in Oxford) and that of Prof. Dimitar Nikolov (at the Sloan Kettering Institute, New York) contemporaneously determined crystal structures of the semaphorin sema domain [10,11], the hallmark N-terminal domain implicated in plexin recognition. The vertebrate semaphorins are grouped, based on ectodomain sequence, into the secreted class 3 semaphorins (the Sema3s), and the cell attached (by single transmembrane helix or GPI anchor) semaphorin classes 4, 5, 6 and 7. Our crystal structure of the human Sema4D ectodomain (hSema4D_ecto_) [10] revealed a seven-bladed β-propeller (the sema domain), a cysteine-rich knot (the PSI domain) and an Ig-like β-sandwich domain. The β-propeller topology, commonly found in extracellular and cytosolic proteins, comprises a series of four-strand anti-parallel β-sheets (the blades) arrayed sequentially around a central axis and locked into full circle by an N-terminal β-strand providing the outermost component of the C-terminal blade (figure 2*a*). The sema domain β-propeller topology is distinguished by an elaborate insertion (of some 70 residues between β-strands C and D of blade 5) that we termed the extrusion [10]. The first crystal structures also revealed the homodimeric architecture of the semaphorins [10,11]. The key factor mediating the dimerization is the sema domain; the top surfaces of the β-propellers abut, off-centre, to form a ‘face-to-face’ interface (figure 2*a*). In total, there are now crystal structures for class 3, 4, 6 and 7 semaphorins [10–15]. All show the same dimeric architecture, posing the question how might this property of the sema domain contribute to function?

## 4. The plexins

Semaphorins typically trigger signalling by interaction with members of the plexin family of cell surface receptors [5]. The plexins are type 1 single membrane spanning glycoproteins with substantial N-terminal ectodomains and C-terminal cytoplasmic regions. Sequence analysis indicates that, as in the semaphorins, the N-terminal portion of the plexin ectodomain has the topology of a sema domain [5,16]. This observation prompted a variety of hypotheses for the role of sema–sema-type interactions in the initiation of semaphorin signalling [11,17,18]. Is the homodimeric sema–sema interface of the semaphorin dimer replaced in a heterodimeric interaction between semaphorin and plexin sema domains? Conversely, does the interaction with semaphorin ligand disrupt an autoinhibitory interaction between the sema domains of unliganded plexin receptors? A combination of structural and functional studies was clearly the way forward to resolve such questions; however, until recently the structures of the plexin ectodomain and semaphorin–plexin complexes resisted characterization. Plexins subdivide into four classes, A, B, C and D. We set out to determine structures of representative members for both major (A and B) classes of plexins in complex with their cognate semaphorin ligands.

## 5. The challenges of glycoprotein sample preparation

The complex, cysteine-rich architectures of glycoprotein ligands such as the semaphorins, or the ectodomains of receptors such as the plexins, are not conducive to high-level expression in *Escherichia coli*. In 2003, to produce the protein sample for our first crystal structure of a semaphorin ectodomain, hSema4D_ecto_, we engineered stable high level expression in mammalian (CHO) cells [10]. The generation and selection of cells giving high level expression of a single hSema4D_ecto_ construct took several months. Over the past decade we have developed, and refined, a transient expression system which uses mammalian cells (typically HEK293 cells) to produce, within weeks rather than months, secreted glycoproteins for structural and biophysical studies [19]. This system allows us to investigate the properties of multiple constructs very efficiently, a strategy we frequently need to implement in order to identify a functional form of our protein or complex of interest that is suitable for high-resolution structure determination. The extracellular regions of proteins typically exhibit substantial amounts of glycosylation. These sugar moieties introduce elements of heterogeneity in terms of molecular composition and structural flexibility that are often deleterious for crystal growth. To circumvent these problems, we are able to exploit strategies, pioneered in Oxford by my colleague Prof. Simon Davis, to manipulate the nature and level of the N-linked glycosylation on mammalian cell expressed proteins using either the glycosylation inhibitor kifunensine or the mutant cell line HEK293S GnTI^−^ [20,21]. We have also developed methods that, when necessary, allow us to label the expressed proteins with selenomethionine to facilitate phasing [19]. Equipped with these methodologies, we successfully expressed secreted forms of murine plexin A2 and one of its semaphorin ligands, murine Sema6A, as well as of the cognate receptor for our hSema4D_ecto_, human plexin B1, for structural and functional studies [12].

## 6. The contribution of Diamond to our understanding of semaphorin function

The capabilities of third-generation synchrotron beam lines have played a critical role in structure determinations of semaphorins, of the semaphorin-binding region of plexin ectodomains and of semaphorin–plexin complexes. For work in my laboratory, the macromolecular crystallography beamlines at the Diamond Light Source have allowed a succession of structure determinations to be brought to successful conclusions despite the challenges presented by the nature of our glycoprotein targets.

A construct comprising essentially the entire ectodomain of the mouse Sema6A (sema domain plus one PSI domain; mSema6A_ecto_) crystallized and diffracted to 2.3 Åresolution at Diamond beamline I03. The structure, one semaphorin dimer of 145 kDa per asymmetric unit, showed the classical sema domain topology and dimeric structure [12] (figure 2*a*). Sequence analysis of plexin ectodomains indicates a highly conserved architecture comprised of an N-terminal sema domain, followed by a combination of three cysteine knot and six β-sandwich type folds, termed PSI and IPT domains, respectively. A construct comprising the sema domain, PSI, IPT and second PSI domain of murine plexin A2 (mPlxnA2_1−4_) crystallized and we collected a 2.3 Åresolution X-ray diffraction dataset at the European Synchrotron Radiation Facility beam ID23-1. In parallel, we grew crystals of the mPlxnA2_1−4_–mSema6A_ecto_ complex. These crystals required extensive optimization, and it was necessary to deglycosylate both mPlxnA2_1−4_ and mSema6A_ecto_. Ultimately, the complex crystals diffracted to 2.2 Å resolution at Diamond I03. These data were phased by molecular replacement using as search models the newly determined mSema6A_ecto_ structure and the distantly related Met-receptor structure (PDB code 2UZX). After several cycles of refinement and manual rebuilding, the electron density for mPlxnA2_1−4_ in the complex structure was used successfully to phase the uncomplexed structure revealing one plexin monomer of 85 kDa per asymmetric unit. In order to provide a representative structure of a semaphorin–plexin interaction involving a class B plexin, we then crystallized and collected data (Diamond I03) for hSema4D_ecto_ in complex with the two N-terminal domains of human plexin B1 (hPlxnB1_1−2_).

Our synchrotron X-ray diffraction data had yielded crystal structures for the individual semaphorin ligands, a plexin receptor and two approximately 300 kDa ligand–receptor complexes. These structures allowed us to address some of the fundamental questions concerning the atomic-level mechanism of semaphorin–plexin signalling.

## 7. The sema domain of the plexins is monomeric

The crystal structure of mPlxnA2_1−4_ (figure 2*b*) reveals that, as expected from sequence analysis, the N-terminal domain of the plexin ectodomain also conforms to the seven-blade β-propeller topology of the sema domain and again includes the distinctive elaboration that we have termed the extrusion [12]. Sequence analyses across the human genome detect sema domains in only three families: firstly the semaphorins, secondly the plexins and thirdly the family of proteins comprising the receptor tyrosine kinases MET and RON (reviewed in [22]). Detailed structural comparison reveals that the plexin sema domain is more closely related to that of the MET receptor than to the prototypic sema of the semaphorins [12]. Crystal structures of MET receptor ectodomain fragments, in complex with either a domain of the physiological ligand hepatocyte growth factor/scatter factor or the bacterial protein internalin B, show no homodimeric arrangement of MET sema domains [23,24]. Likewise, our crystal structure of mPlxnA2_1−4_ is monomeric. It appears that unlike its counterpart in semaphorins the plexin sema domain shows little or no propensity to homodimerize. We and others have been unable to detect any measureable interactions (at least to *K*_d_ values more than 300 μM) in biophysical analyses of secreted plexin constructs comprising sema–PSI or sema–PSI1–IPT1–PSI2 [12,13].

Overall, our mPlxnA2_1−4_ crystal structure provides information on four domains from what is in total a 10 domain ectodomain. It reveals a relatively elongated structure comprising the N-terminal sema domain followed by the three domains of the PSI1–IPT1–PSI2 region. These three domains are arranged sequentially, pointing away from the sema domain, and exhibit the predicted cysteine knot and β-sandwich type folds (figure 2*b*).

## 8. What happens when semaphorin meets plexin?

To recapitulate, the semaphorin ectodomain dimerizes through a sema-to-sema domain interaction in all crystal structures of the isolated ligands determined to date [10–15]. This is highly indicative of the homodimer being the physiologically functional form for this cell guidance cue. This conclusion is further supported by biophysical measurements: for example, in our hands the mSema6A_ecto_ dimer appears stable in analytical ultracentrifugation and multi-angle light scattering measurements, consistent with the extensive dimer interface (2600 Å^2^ buried surface area) present in the crystal structure [12]. What then is the stoichiometry and architecture of plexin–semaphorin recognition? The crystal structures for mPlxnA2_1−4_–mSema6A_ecto_ and hPlxnB1_1−2_–hSema4D_ecto_ we determined using X-ray diffraction data collected on Diamond beamlines allowed us to address this question. These structures revealed semaphorin–plexin complexes for both classes of plexin comprise the dimeric semaphorin interacting with two essentially separate plexin molecules through side-to-face binding of the sema domains (figure 2*c*). Each side-to-face interaction between semaphorin and plexin sema domains buries a surface area of 2000–2500 Å^2^ and requires no major conformational change in either the semaphorin or the plexin. The extensive interface is mediated by residues which show high levels of sequence conservation within the vertebrate classes of semaphorins and plexins, thus it appears that our complex structures reveal an architecture that is generic for this signalling system. However, there are significant variations in the detailed structures of the mPlxnA2_1−4_–mSema6A_*ecto*_ and hPlxnB1_1−2_–hSema4D_ecto_ interfaces consistent with the specificity of these particular ligand–receptor pairings.

## 9. Semaphorin bivalency is necessary to trigger plexin signalling

We used surface plasmon resonance (SPR) equilibrium binding studies to demonstrate that the bivalent nature of the semaphorin interaction can result in a substantial (up to 50-fold), avidity-based, increase in the binding affinity (*K*_d_ of 2.5 μM for the monomeric mPlxnA2_1−4_ binding to a mSema6A_ecto_-coated surface, independent of the coating density; *K*_d_ of 0.048 μM for the dimeric mSema6A_ecto_ binding to a surface densely coated with mPlxnA2_1−4_ versus a *K*_d_ of 0.28 μM for a more sparsely coated surface). Structure-guided design allowed us to generate mutant forms of the semaphorin ectodomains which were monomeric. These mutant molecules were stable and were still capable of binding plexin, but showed no avidity effect in SPR measurements. Significantly monomeric Sema4D_ecto_ could no longer elicit a response in the standard cellular assay for plexin B1 signalling, the cell collapse assay [12].

## 10. Atomic-level details of semaphorin–plexin recognition and the wiring of the brain

We were also able to combine structure-guided mutagenesis with SPR and cell collapse assays to verify the physiological relevance of the semaphorin–plexin interface observed in our crystal structures [12]. Indeed, the crystal structure of the mPlxnA2_1−4_–mSema6A_*ecto*_ complex provides insight, at the atomic level, into changes, which others have reported, in the organization of the cerebellum of a mutant mouse. The single nucleotide substitution of cytosine by adenine at position 1187 of the *Plxna2* gene in an ENU mutagenesis screen of C57BL6/J mice has been found to effect the migration of granular cells leading to a failure of this population of neuronal cells to segregate to the correct layer of the cerebellum [25]. This single nucleotide substitution results in replacement of alanine (396) by glutamic acid (A396E) in the mPlxnA2 protein, a change in a surface residue of the plexin sema domain which the crystal structure shows to be directly involved in the interaction with mSema6A_ecto_. We used SPR binding measurements of mSema6A_ecto_ and an A396E mutant mPlxnA2_1−4_ to confirm there is essentially complete loss of binding affinity. The change in the architecture of the mutant mouse brain is the result of a difference of a few atoms in an amino acid side chain causing loss of recognition between plexin receptor and semaphorin ligand. The cell guidance interaction is abolished.

## 11. Further levels of complexity, enter the co-receptor

There are 19 members of the five semaphorin classes in humans as opposed to nine members of the four classes of plexins. Consistent with the mismatch in the number of distinct ligands and receptors there is substantial crossreactivity. This promiscuity is particularly striking for the class A plexins which are known to serve as the signalling receptors for multiple members of the class 3 and class 6 semaphorin ligand families (reviewed in [7,26]). The class 3 semaphorins are the only class of semaphorins in vertebrates that are not tethered to the cell surface. These ligands were the first of the vertebrate semaphorins to be characterized [3] and were first shown to associate with the neuropilin family of cell surface receptors [27,28]. Although it has subsequently emerged that the class A plexins are required to transduce class 3 semaphorin signals, cell-based assays have revealed no evidence for a direct interaction between these semaphorin ligands and their plexin receptors; instead the interaction requires a holoreceptor comprising plexin and neuropilin [5,29]. To address the conundrum of Sema3A signalling we turned again to the Diamond Light Source which played a crucial role, allowing us to collect X-ray diffraction data from particularly challenging crystals.

## 12. The semaphorin–neuropilin–plexin complex

The neuropilins, neuropilin 1 and 2 (Nrp1 and Nrp2), are type 1 cell surface receptors with ectodomains comprising five distinct domains, a single transmembrane span, and a short, unstructured, cytoplasmic region. Class 3 semaphorin binding has been reported to require the N-terminal four domains of the neuropilins [11,30–33]; these comprise two CUB domains (termed a1 and a2) and two coagulation factor V/VIII homology domains (termed b1 and b2). A crystal structure of this four domain region (in complex with a Fab fragment) has been determined for human Nrp2 [33]. We demonstrated in a series of SPR binding assays [15] a direct interaction between the a1–a2–b1–b2 region in mouse Nrp1 (mNrp1_1−4_) and our previously characterized mPlxnA2_1−4_. We also detected binding between mSema3A_S−P_ (a form of mouse Sema3A comprising the sema and PSI domain) and mNrp1_1−4_ but, in agreement with all the previously reported cell-based studies, found no measurable binding between mSema3A_S−P_ and mNrp1_1−4_. On combining mSema3A_S−P_ and mNrp1_1−4_, we could show an additive effect for interaction with mPlxnA2_1−4_.

**Figure 3.**
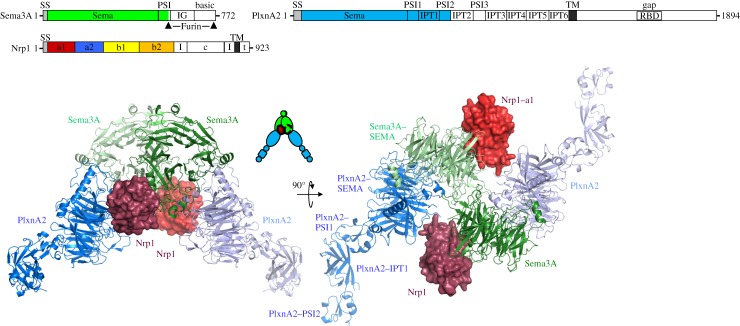
The semaphorin–neuropilin–plexin complex. The schematic domain organization of mouse Sema3A, PlxnA2 and Nrp1 is presented above two orthogonal views of the structure of a semaphorin–neuropilin–plexin complex. Sema3A sema domain (green) and PlxnA2_1−4_ (blue) structures are shown in ribbon representation and Nrp1 domain a1 structures (red) in surface representation. (Adapted from [15].)

We determined a crystal structure of mNrp1_1−4_ (2.7 Åresolution; Diamond I04-1) [15], and so had high-resolution structures in hand for all the components of the semaphorin–neuropilin–plexin complex (mPlxnA2_1−4_ at 2.3 Åfrom our earlier studies [12], and a 2.8 Åsema domain dimer structure for mSema3A from the laboratory of Dimitar Nikolov [11]). These well-characterized individual structures proved essential for our structure determination and analysis of the complex [15]. After extensive crystal optimization and screening, the final diffraction dataset we were able to generate for the semaphorin–neuropilin–plexin complex extended to 7 Å resolution and was the result of merging the diffraction data collected from three crystals, one each on Diamond beamlines I04-1, I02 and I03. The crystal structure was solved by molecular replacement revealing an asymmetric unit containing six copies of PlxnA2_1−4_ and one copy each of the Sema3A sema domain and Nrp1_1−4_ (Nrp1 domains a2, b1 and b2 were omitted from the model because of disorder). Given the low resolution of the diffraction data, the model was only subjected to rigid-body refinement, with each domain treated as a rigid group (26 groups in total: four for each PlxnA2 molecule and one each for Sema3A and Nrp1) and a single B factor per domain. Twofold crystallographic symmetry generates the biologically relevant 330 kDa complex which comprises the semaphorin dimer, two neuropilin a1 domains and two plexins (figure 3).

Our semaphorin–neuropilin–plexin crystal structure reveals that the arrangement of dimeric semaphorin separately interfacing two plexin receptors is identical to the architecture we, and others, have found in all other semaphorin–plexin complexes [12–14]. So how does the neuropilin co-receptor stabilize this generic architecture given that the semaphorin–plexin binding affinity is undetectable for Sema3A to PlxnA2? The crystal structures of a1–a2–b1–b2 Nrp1 and Nrp2 [15,33] point to the a1 domain being flexibly linked to the tightly clustered a2–b1–b2 unit. Only the Nrp1 a1 domain contributes to the complex in our Sema3A–Nrp1–PlxnA2 crystal structure; however, the role of this domain is crucial. It serves to bridge between the sema domain of one semaphorin subunit and the sema domain of the plexin bound to the second semaphorin subunit [15] (figure 3). Thus, the a1 domains of two neuropilin co-receptors cross-brace the semaphorin–plexin complex. Previously reported functional data as well as our own biophysical and cellular analyses of mutant proteins are consistent with the Sema3A–Nrp1, Nrp1–PlxnA2 and Sema3A–PlxnA2 interfaces we observe for the semaphorin–neuropilin–plexin complex [15]. Indeed, an earlier observation, by others, that Sema3A^K108N^ mutant mice show severe defects in the development of their peripheral nervous system [34] is now explained by the involvement of this residue in the semaphorin–plexin interface.

## 13. Semaphorin–plexin signalling: current understanding and remaining questions

In this review, I have focused on the results which now form the basis of our understanding of the structure and function of the extracellular components of the semaphorin–plexin system. These results highlight the key role played by synchrotron radiation in enabling structural studies of challenging glycoprotein complexes. In summary, our structural and functional data suggest a model in which the bivalent interaction of the semaphorin ligand with two plexin receptors provides an essential trigger for cell guidance signalling. For the secreted class 3 semaphorins, the co-receptor neuropilin is necessary to cement together this core complex. Further work can now build on these insights to probe the architecture and role of the entire plexin ectodomain, the requirements for signal transduction, and whether the formation of the initial semaphorin–plexin interface generates a standalone signalling complex or provides the first step in a larger-scale clustering of receptors.

We and others have also made progress in the analysis of the molecular mechanisms controlling plexin signal transduction within the cell (reviewed in [35]). Again, structural studies using synchrotron beamlines have paved the way for advances in understanding. For example, structural analyses carried out in my laboratory, and independently in the laboratory of Prof. Mario Amzel (Johns Hopkins University), revealed distinctive features in the substrate binding site of MICAL, a multi-domain flavoenzyme-signalling molecule implicated in plexin A signal transduction [36,37], observations which led us to suggest that the monooxygenase activity of this flavoenzyme was targeted to hydroxylation of a protein side chain, most probably in actin [36]. Subsequent studies have duly demonstrated that MICAL can oxidize two specific methionine residues in actin and hence destabilize F-actin filaments for plexin-mediated cell repulsive effects [38]. How does the plexin regulate MICAL activity? At present, we do not know the answer to this question. The details of the interaction between MICAL and the plexin cytoplasmic region are as yet uncharacterized. Indeed, many questions remain open regarding the mechanism(s) of action of the plexin cytoplasmic region [35]. Studies by us and others have revealed the topology to be that of a classical Ras GTPase activating protein (GAP) domain (albeit a GAP domain fold which is interrupted by the insertion of some 120 residues which form a discrete Rho GTPase-binding domain) [39–41]; however, recent data have provided compelling evidence of its activity as a GAP for Rap rather than as Ras homologues [42]. Clearly, much work remains to be done to illuminate fully the molecular mechanisms of plexin signalling.

## 14. Conclusion: paving the way for new therapeutics?

Semaphorin–plexin-mediated cell guidance was first characterized in the nervous system, but is now known to function in the development and homeostasis of myriad tissues and organs. This broad swath of biology includes numerous areas in which semaphorin–plexin signalling has emerged as a potential target for therapeutic intervention. For example, the recent finding that Sema3A acts as a biological regulator of bone homeostasis, able both to reduce bone resorption and increase bone synthesis, is of potential importance for the development of therapeutics to combat osteoporosis [43]. As a result of its role in the regulation, activation and migration of inflammatory cells, Sema4D is the target of a therapeutic antibody (VX15/2503, Vaccinex Inc.) currently in phase I clinical trials for patients with multiple sclerosis (ClinicalTrials.gov identifier: NCT01764737). The broadest area for potential therapeutic intervention is that of cancer biology (reviewed in [44]) where multiple members of the semaphorin family (as well as their plexin receptors and neuropilin co-receptors) have been found to be aberrantly expressed in human tumours. In one recent example, in a genomic characterization of pancreatic ductal adenocarcinoma (the major type of pancreatic cancer) high expression of Sema3A and PlxnA1 was found to co-segregate with poor patient survival [45]. However, even for a single family member, such as Sema3A, an overall survey of the literature reveals data consistent with both tumour suppressive and tumour promoting roles [44]. Our ability to design therapeutic strategies to manipulate semaphorin–plexin signalling will require that we dissect out the underlying molecular mechanisms of this multi-faceted system. We are fortunate in having the facilities of the Diamond Light Source to help in our further exploration of this complex system.
